# Intestinal Health and Threonine Requirement of Growing Pigs Fed Diets Containing High Dietary Fibre and Fermentable Protein

**DOI:** 10.3390/ani10112055

**Published:** 2020-11-06

**Authors:** Michael O. Wellington, Rochelle B. Thiessen, Andrew G. Van Kessel, Daniel A. Columbus

**Affiliations:** 1 Prairie Swine Centre, Inc., Saskatoon, SK S7H5N9, Canada; michael.wellington@usask.ca (M.O.W.); rochelle.thiessen@usask.ca (R.B.T.); 2 Department of Animal and Poultry Science, University of Saskatchewan, Saskatoon, SK S7N5A8, Canada; andrew.vankessel@usask.ca

**Keywords:** dietary fibre, fermentable protein, protein deposition, threonine, gut health

## Abstract

**Simple Summary:**

Dietary components, such as fibre and protein, have significant impacts on nutrient requirements and intestinal health in pigs. The objectives of this study were to investigate the interactive effects of dietary fibre and fermentable protein on threonine requirement for protein deposition in growing pigs and to determine how these factors affect markers of intestinal health. In this study we used the nitrogen-balance approach to study the influence of high protein diets combined with high fibre on threonine requirement for protein deposition. We further used gene expression, fermentation metabolites (short and branched chain fatty acid concentration), and serum antioxidant status in these pigs as markers of intestinal health and function. We demonstrate that high fibre will indeed increase threonine requirement for protein deposition but can mitigate the negative effects of fermentable protein metabolites on intestinal health. These results will have implications for the development of dietary strategies to improve growth and overall health in pigs, including adjustments to dietary fibre, protein, and amino acid content that maximize pig growth, nutrient utilization, and intestinal health.

**Abstract:**

Dietary fibre (DF) and fermentable crude protein (fCP) are dietary factors which affect nutrient utilization and intestinal health in pigs. A nitrogen (N)-balance study was conducted to determine the impact of DF and fCP on threonine (Thr) requirement for protein deposition (PD) and indicators of intestinal health. A total of 160 growing pigs (25 kg) were randomly assigned to 1 of 20 dietary treatments in a 2 × 2 × 5 factorial arrangement in a randomized complete block design with dietary fibre (low (LF) or high fibre (HF)], fCP [low (LfCP) or high fCP (HfCP)) and Thr (0.52, 0.60, 0.68, 0.76, or 0.82% standardized ileal digestible) as factors. Then, 4-day total urine and fecal collection was conducted, and pigs were euthanized for intestinal tissue and digesta sampling. Feeding high DF, regardless of fCP content, increased Thr requirement for PD (*p* < 0.05). High fCP, regardless of DF content, reduced Thr requirement for PD. Serum antioxidant capacity increased as dietary Thr level increased (*p* < 0.05). Cecal digesta short-chain fatty acids (SCFA) increased (*p* < 0.05) with HF and branched-chain fatty acids (BCFA) increased with HfCP and reduced with HF (*p* < 0.05). HfCP reduced (*p* < 0.05) mucin-2 (MUC2) expression in the colon of the HF but not the LF fed pigs and HF increased MUC2 in the LfCP but not the HfCP fed pigs. Feeding HF diet increased (*p* < 0.05) expression of zonula occludens-1 in the LfCP with no effect on HfCP fed pigs. Ammonia concentration in both cecum and colon increased (*p* < 0.05) in the HfCP fed pigs. Overall, high DF reduced the negative impact of HfCP on intestinal health, as indicated by alterations in SCFA and BCFA production and gut barrier gene expression. While increased dietary Thr content is required for PD in pigs fed high DF, feeding high fCP reduced Thr requirements.

## 1. Introduction

Combining different feedstuffs to formulate swine diets is essential for the supply of amino acids (AA), energy, and other nutrients necessary for optimal growth and nutrient utilization and production. While the main goal of diet formulation is meeting nutrient requirements, the impact different feedstuffs have on nutrient utilization (e.g., nitrogen retention) and intestinal physiology (e.g., gut health) of the pig also need to be considered. For instance, dietary components, such as dietary fibre (DF) and crude protein (CP), influence nutrient use and intestinal health. Mechanisms for these effects include reduced nutrient availability and microbial metabolite production [1,2] modulation of gut microbiome [3], and changes in intestinal morphology [4]. For example, DF can reduce nutrient digestibility [5,6] and increase endogenous AA losses [7], resulting in greater threonine (Thr) requirement for protein deposition (PD) and growth [2,8,9].

On the other hand, the inclusion of high-DF feedstuffs in swine diets can positively affect intestinal health, promoting the growth of beneficial bacteria and production of metabolites (e.g., short-chain fatty acids) [10]. Feeding high-CP diets has been reported to negatively affect weanling pigs [11] due to an increase in the amount of protein flow to the hind gut (i.e., fermentable protein (fCP)), promoting growth of pathogenic bacteria and production of harmful metabolites. Metabolites of protein fermentation (e.g., ammonia, amines, phenolic and indole compounds) have been associated with a negative impact on gut health and predisposition to post-weaning diarrhea following enterotoxigenic *E. coli* challenge [12]. Although feeding high DF increases endogenous Thr losses [13] and increases dietary Thr requirement for growth and PD [2,9], the inclusion of DF in swine diets has been suggested as a potential mechanism to mitigate the negative effects of protein fermentation [14] by providing gut microbes a preferential fermentation substrate. For example, inclusion of DF has been reported to reduce ammonia and putrescine levels in pigs fed a diet containing highly fermentable protein [14]. The reduction in protein fermentation has been suggested to not only be dependent on the provision of alternative fermentable substrates, such as DF, but also the fibre type (e.g., soluble fibre vs. insoluble fibre) and site of fermentation [15].

The objective of the present study was to investigate the interactive effects of DF and fCP on Thr requirement for PD and further elucidate how these factors affect markers of intestinal health. Based on the associated effects of fCP on the gut and impact on nutrient utilization, we hypothesized that feeding high DF and high fCP will interactively increase the Thr requirement for PD and to maintain intestinal health and barrier function.

## 2. Materials and Methods

### 2.1. Ethical Statement

The experimental protocol was reviewed and approved by the University of Saskatchewan’s Animal Research Ethics Board (#20130054) and followed the approved animal care guidelines.

### 2.2. Animals, Housing, Diets, and Experimental Design

A total of 160 growing pigs (Camborough Plus × C3378: PIC Canada Ltd., Winnipeg, MB, Canada) with initial body weight (BW) of 19.5 ± SD = 1.05 kg were used in a nitrogen (N)-balance experiment at the Prairie Swine Centre, Inc. (Saskatoon, Canada). The pigs were individually housed in metabolism crates (1.4 × 1.5 m) in a temperature-controlled room (20 ± 2 °C) and randomly assigned to 1 of 20 dietary treatments in 8 blocks (room) (*n =* 8). The experiment was conducted as a randomized complete block design arranged as a 2 × 2 × 5 factorial of the dietary treatments. The factors were dietary fibre content (low fibre (LF; 13% total DF) or high fibre (HF; 20% total DF)), fermentable protein content (low fermentable crude protein (LfCP; 16% CP) or high fermentable crude protein (HfCP; 18% CP)), and dietary Thr content (0.52, 0.60, 0.68, 0.76, and 0.82% standardized ileal digestible [SID] Thr). Dietary Thr levels were based on previous studies using similar diet composition [2,9] which estimated a requirement of 0.68% SID Thr for maximum PD in pigs of the same population, age, and BW. The diets were formulated to meet or exceed nutrient requirements according to NRC [16], except for Thr, and contained celite as an indigestible marker. The HF diet was formulated by adding 10% sugar beet pulp and 5% wheat bran at the expense of corn in the LF diet based on previous studies [2,9,14]. The HfCP diets were achieved by the addition of soybean meal that had been autoclaved at 124 °C for 20 min (10 kg batches of soybean meal were evenly spread on a tray to allow for equal heat distribution to the sample), which has been shown previously to reduce the digestibility of protein and AA [14,17], increasing the flow of protein into the hindgut. As shown in Table 1, the highest and lowest Thr levels in each DF and fCP level were formulated and blended in appropriate proportions to achieve the 5 levels of Thr within each dietary treatment group (*n* = 8). Daily feed was offered at 2.8 × maintenance metabolizable energy requirements in two equal meals at 0800 h and 1500 h with ad libitum access to water. Feed refusals were collected for each pig daily and weighed to ascertain actual daily feed intake. The experiment lasted a total of 12 day which consisted of an 8-day adaptation period and a 4-day collection period with daily fresh fecal grab samples and daily total urine collection.

### 2.3. Nitrogen-Balance, Blood, Digesta, and Tissue Sampling

During the 4-day collection periods, fresh fecal grab samples were collected daily by rectal palpation and stored at −20 °C. At the end of each collection period, fecal samples were thawed and pooled for each pig and homogenized. Subsamples were collected and stored at −20 °C until analysis. Total urine samples were collected quantitatively daily over a 24-h period for each pig using metal trays and jars placed underneath the metabolism crates. The urine collection jars contained approximately 20 mL of 6 N HCl to reduce nitrogen losses. At the end of each collection day, total urine was weighed and sub-sampled per pig. All urine sub-samples were then pooled for each pig over the 4-day collection period and stored at −20 °C until further analysis. On the day following the N-balance collection period, blood samples were taken via jugular puncture from all pigs 3 h after the morning meal into vacutainer tubes (BD, Vacutainer tubes). The samples were centrifuged at 2500× *g* for 15 min and the serum was collected and stored at −20 °C for analysis of serum antioxidant capacity. Following blood sampling, pigs fed the 0.52%, 0.68%, and 0.82% SID Thr diets were euthanized by penetrating captive bolt followed by exsanguination. Colonic tissue samples were collected at the ileum (30 cm from ileo-cecal junction), cecum, and apex of the colon, immediately snap frozen in liquid nitrogen, and stored at −80 °C for qPCR analysis. Digesta samples were collected from the caecum and colon into 15 mL tubes, snap frozen in liquid nitrogen, and stored at −80 °C for ammonia and short chain (SCFA) and branched chain (BCFA) fatty acid analysis.

### 2.4. Feed and Fecal Analysis

The diet DM content was analyzed according to the Association of Official Analytical Chemists (AOAC) method 930.15 [18]. Dietary N content was analyzed using an automatic N analyzer (LECO FP 528; MI, USA; method 990.03) [18] and CP content was determined as N × 6.25. Total dietary fibre (TDF), soluble dietary fibre (SDF), and insoluble dietary fibre (IDF) of the complete diets were analyzed according to the AOAC [18] method 991.43 using an ANKOM^TDF^ dietary fibre analyzer (ANKOM Technology, Macedon, NY, USA). The analyzed nutrient composition of the experimental diets is presented in Table S1. Fecal samples were freeze-dried before grinding in a centrifugal mill (ZM 100, RETSCH GmbH & Co., Haan, Germany) to pass through a 1-mm sieve. Dry matter of fecal samples and N content of urine and fecal samples and diet samples were analyzed. Acid-insoluble ash content of the diets and fecal samples were measured according to the prescribed protocol [19]. Nitrogen retention was calculated as the difference between total N intake (dietary N) minus total N output (fecal and urinary N). Protein deposition was calculated as total N retained × 6.25.

### 2.5. Serum and Digesta Analysis

Serum samples were analysed for total antioxidant capacity using a commercially available kit (#CS0790; Sigma Aldrich, Oakville, Canada) according to the manufacturer’s instructions. Cecal and colonic digesta samples were analysed for ammonia concentration via colorimetry according to a previously described method [20] with modifications. Briefly, phenol and hypochlorite reagents were prepared according to established protocols, following which the digesta samples were thawed, centrifuged at 14,000× *g* for 10 min, and the supernatant kept on ice. In a new test tube, 25 µL of supernatant was added, followed by 1.25 mL of the phenol/hypochlorite reagent and samples were vortexed before adding 1 mL of the hypochlorite reagent after which samples were vortexed and placed in a 95 °C water bath for 5 min. Samples were then placed in a cold-water bath for 3 min after which 2.5 mL distilled water was added to each sample and vortexed. Samples were then transferred into cuvettes and analyzed using a spectrophotometer (Helios Delta, Thermo Fisher Scientific, Waltham, MA, USA) at 630 nm wavelength. Short-chain and branched-chained fatty acids were analysed in cecal and colonic digesta following the procedure of Khorasani et al. [21] and Lenahan et al. [22]. Briefly, digesta samples were diluted with 25% metaphosphoric acid at a 2:1 ratio (*w*/*v*). Samples were centrifuged at 12,000× *g* for 10 min and the supernatant was collected in 2-mL centrifuge tubes and centrifuged at 16,000× *g* for 10 min. The supernatant was then collected and filtered into 1.5-mL tubes. An internal standard, isocaproic acid was added at 0.2 mL to 1 mL of the filtered sample supernatant and inverted to mix thoroughly. Samples were aliquoted into glass vials for analysis in the gas chromatograph equipped with a flame ionization detector (Agilent Technologies, Santa Clara, CA, USA).

### 2.6. Gene Expression Analysis

Tissue samples from the ileum, cecum, and colon were analyzed for pro- and anti-inflammatory cytokines (IL-1β, IL-10, IL8), tight junction protein (ZO-1, CLDN4), gut function (PCNA, Casp3), and mucin (MUC2, MUC5AC) transcript abundance using GAPDH and RPL19 as the housekeeping genes to normalize the expression of the target genes (Table S2). Briefly, total RNA was extracted from tissue samples using TRIzol reagent (Invitrogen, Carlsbad, CA, USA) according to the manufacturer’s protocol. The RNA concentration and quality were determined using a spectrophotometer (NanoDrop 2000, Thermo Fisher Scientific) with an optical density ratio (260:280) between 1.8 and 2.0. Reverse transcription was carried out on the RNA samples using high capacity cDNA reverse transcription kit (Applied Biosystems, Waltham, MA, USA) with random hexamer. The PCR reactions were carried out in 96-well plates. Each reaction mix made a total volume of 20 µL which contained 0.8 µL each of appropriate forward and reverse primers, 6.4 µL of nuclease-free water, 10 µL of EVA Green supermix (Bio-Rad Laboratories, Hercules, CA, USA), and 2 µL (2 ng/PCR reaction) of template cDNA. Standard curves were made for each gene using a 5-fold serial dilution of pooled cDNA samples from all experimental treatments. PCR efficiency output between 90% and 110% were accepted and included in analysis. The final analysis was done with a standard dilution series prepared from cDNA on the same plate and the starting quantities (recorded as arbitrary units) were calculated for each gene on each plate.

### 2.7. Statistical Analyses

All data were tested for normality using the studentized residual analysis and values which were 3 standard deviations from the treatment mean were identified as outliers and removed (Shapiro–Wilk test; PROC UNIVARIATE; SAS 9.4). For the N-balance and serum antioxidant data, analysis was completed as a 2 × 2 × 5 factorial arrangement with fibre level (*n* = 2; fixed effect), fermentable protein level (*n* = 2; fixed effect), Thr level (*n* = 5; fixed effect), and their interactions included in the model as fixed effect with block (room) (*n = 8*) as a random effect. For all other data, the analysis was completed as a 2 × 2 × 3 factorial arrangement with fibre level (*n* = 2; fixed effect), fermentable protein level (*n = 2;* fixed effect), and Thr level (*n* = 3; fixed effect). The best fitting model based on the coefficient of determination (R^2^) was the quadratic breakpoint (PROC NLIN; SAS 9.4). The model was used to estimate Thr requirements in LF and HF diets with LfCP or HfCP content. Significance was defined as *p* < 0.05 and a trend towards significance was considered at *p* ≤ 0.10. Significant means were separated by the Tukey–Kramer test.

## 3. Results

### 3.1. Protein Deposition and Threonine Requirement

**Figure 1:**
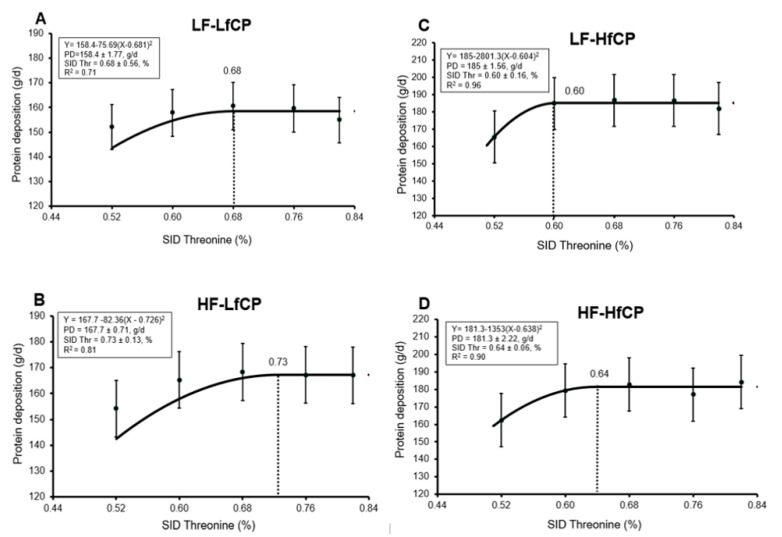
Quadratic breakpoint model analysis of threonine requirement in growing pigs fed a diet with low (LF) or high (HF) fibre content and low (LfCP) or high (HfCP) fermentable protein content (n = 8/treatment). The breakpoint estimate for LF-LfCP was 0.68% standardized ileal digestible (SID) Thr for maximum protein deposition (PD) of 158.4 g/d (**A**), HF-LfCP was 0.73% SID Thr for maximum PD of 167.7 g/d (**B**), LF-HfCP was 0.60% SID Thr for maximum PD of 185 g/d (**C**), and HF-HfCP was 0.64% SID Thr for maximum PD of 181 g/d (**D**). Each data point represents and average protein deposition value (*n* = 8).

Nitrogen-balance data are presented in Table 2. There was a significant interaction between DF and fCP level (*p* < 0.01) on N intake, such that N intake was higher in pigs fed both the HfCP and HF diets. Similarly, a significant interaction (*p* = 0.04) between Thr and fCP was observed on N intake, where pigs fed the HfCP had increased N intake as dietary Thr increased. The urinary and fecal N output were both affected (*p* < 0.05) by a DF and fCP interaction. Specifically, pigs fed the HfCP diets had increased urinary N output, while pigs fed the HF diets had reduced urinary N output. Fecal N output was increased in both HfCP and HF-fed pigs. Apparent total tract digestibility (ATTD) of N was affected (*p* < 0.01) by DF and Thr, but there was no significant effect of fCP. Feeding the HF diets reduced the ATTD of N, while increasing dietary Thr increased the ATTD of N. Protein deposition was affected (*p* < 0.05) by DF × fCP and Thr × fCP interactions. The interaction between DF and fCP was such that HfCP increased PD in the LF but not HF diets. The interaction between Thr and fCP was such that PD was increased in both LfCP and HfCP diets and was also increased in HfCP diets as dietary Thr increased. Dietary Thr requirements for PD, as estimated by the quadratic broken-line model, are presented in Figure 1. The basal Thr requirement, as estimated in pigs fed LF-LfCP, was 0.68% SID (Figure 1A). Within the same LfCP level, the addition of HF increased the estimate of Thr requirement for PD to 0.73% SID (Figure 1B). Within the HfCP diets, estimated Thr requirement for PD with LF-HfCP was 0.60% SID (Figure 1C), while in the HF-HfCP fed pigs, the estimated Thr requirement for PD was 0.64% SID (Figure 1D).

### 3.2. Digesta Ammonia, SCFA, BCFA and Serum Antioxidant Capacity

Digesta SCFA, BCFA, and ammonia concentration in the caecum and colon are presented in Table 3 and Table 4, respectively. The isobutyrate content in the cecal digesta was reduced (*p* < 0.05) in the HF diet, with no significant effects of fCP or Thr content or an interaction. There was a DF × Thr (*p* < 0.05) interaction on butyric acid and isovalerate concentration in cecal digesta. Specifically, butyric acid and isovalerate concentration increased as dietary Thr increased in the HF diet. In the colon, HF diet reduced (*p* < 0.05) isobutyrate and isovalerate concentration. Propionic acid concentration in the colon was affected by the fCP level, specifically, the HfCP diet reduced (*p* < 0.05) propionic acid concentration in the colon digesta, with no significant DF or Thr effects and no interactions. There were no significant treatment effects on the total SCFA concentration in the caecum; however, total BCFA concentration was affected by the DF and fCP level. The HfCP diet increased (*p* < 0.05) total BCFA while the HF diet reduced (*p* = 0.05) total BCFA concentration in the cecal digesta. In the colon, there were no treatment effects on total SCFA and BCFA (data not shown). The ammonia concentration was significantly affected (*p* < 0.05) by DF and fCP levels in both caecum and colon digesta. In both caecum and colon, the HfCP diet increased (*p* < 0.05) while the HF diet reduced (*p* < 0.05) digesta ammonia concentration.

The data on total serum antioxidant status are presented in Table 5. Serum antioxidant capacity was not affected (*p* > 0.05) by DF or fCP level; however, there was a significant effect of dietary Thr content (*p* < 0.05). Specifically, pigs fed a dietary Thr content of 0.60, 0.68, 0.76, and 0.82% SID had greater serum antioxidant capacity compared to the lowest dietary Thr level (0.52% SID).

### 3.3. Gene Expression Analysis

Gene expression of markers of gut health and immune response (IL10, IL8, IL1ß, ZO-1, CLDN4, MUC2 and MUC5ac) were measured in the ileal, cecal, and colonic tissue samples. We observed significant treatments effects only in the colon for specific genes, therefore only those data are presented in Table 6. The HF diet increased (*p* < 0.01) MUC5ac expression, with no significant effect of fCP or Thr levels. For ZO-1 and IL-1β expression, there were significant two-way interactions. There was a DF × Thr interaction (*p* < 0.01), whereby HF, but not LF, increased ZO-1 expression with increasing dietary Thr levels. Additionally, a DF × fCP interaction (*p* < 0.05) was observed where HF increased ZO-1 expression in the LfCP diet but not the HfCP diet. The expression of IL-1β was increased in the LF- HfCP fed group but not in the LF-LfCP fed group (DF × fCP; *p* < 0.01). Increasing dietary Thr levels increased IL-1β expression in the HF-fed group while decreasing expression in the LF-fed group (DF × Thr; *p* < 0.05). The expression of IL-1β was higher in the HfCP-fed group and higher at a dietary Thr content of 0.68% (Thr × fCP; *p* < 0.05). We observed a significant three-way interaction (*p* < 0.01) on MUC2 and IL-10 expression. Increasing dietary Thr in the HfCP group reduced MUC2 expression, while the HF diet increased MUC2 expression in the LfCP group. The expression of IL-10 was reduced as dietary Thr levels increased in the LF fed group but not in the HF-fed group, regardless of the fCP group.

## 4. Discussion

Understanding the effects of dietary components, such as DF and protein, on nutrient requirements and animal health is key to development of dietary strategies to optimize nutrient utilization, growth, and health of growing pigs. Therefore, in the present study we evaluated the independent and interactive effects of DF and fCP on the Thr requirement for protein deposition, largely because Thr has been shown to be important in maintaining gut health and barrier function in pigs [23]. We further evaluated the impact of DF and fCP content on fermentation characteristics and gut health in the pig hindgut. Based largely on results of in vitro studies, protein fermentation products, such as ammonia and BCFA, have been shown to have detrimental effects on the intestinal epithelium largely, with fewer in vivo studies confirming this [24]. As such, dietary strategies aimed at reducing protein fermentation metabolites are thought to improve intestinal health. For example, previous work in pigs showed that when DF is included in high protein diets, protein fermentation and production of harmful metabolites is reduced and there is increased production of beneficial metabolites, such as acetic acid, butyric acid, and propionic acid [14]. Moreover, highly fermentable sources of DF, such as sugar beet pulp, can provide intestinal microbes an alternative, and potentially preferred, source of substrate for fermentation, allowing for the incorporation of N into the microbial biomass, further reducing the concentration of harmful fermentation metabolites (e.g., BCFA, ammonia) [25,26].

To achieve our objective, diets were formulated to vary in DF and fCP content in addition to graded levels of Thr to allow for determination of the effects of diet components on Thr requirement for PD. In the current study, dietary inclusion of fibre and fibre type as well as protein content were based on previous studies examining the impact of DF and fCP on gut health and animal performance [2,9,14]. To achieve the HF diet, we included sugar beet pulp and wheat bran at the expense of corn, a low fibre ingredient. This contrasts with Pieper et al. [14] who added fibre at the expense of wheat, a high fibre ingredient, which resulted in increased insoluble DF content but decreased soluble DF. Our strategy to replace corn in the HF diet with sugar beet pulp and wheat bran ensured that the HF diets had greater levels of both soluble and insoluble DF, which we have reported previously [2,9]. Likewise, while dietary inclusion of soybean meal and dietary protein content was based on Pieper et al. [14], adjustments were made to target values to ensure that the LfCP diet would meet all essential AA requirements and would not limit growth due to AA deficiencies. In the current study, adjustments in ingredient content were made to achieve treatment objectives in DF and fCP content while maintaining similar nutrient content across diets that met or exceeded nutrient requirements, such as energy, amino acids, and protein according to NRC [16]. Inevitably, the choice to use typical feed ingredients, rather than purified diets, to achieve the DF and fCP targets resulted in some unavoidable differences in nutrient content. This is a common feature in nutrition studies, and especially in those examining different DF and fCP content [2,9,14,27]. Moreover, differences in calculated vs. analyzed nutrient content are not unexpected and are largely due to variation in ingredient nutrient content, diet preparation, and laboratory analysis. Results of the current study should be interpreted given these limitations in diet formulation and maintaining consistent nutrient content across diets when varying DF and fCP.

In the current study, the inclusion of high fCP in the diet increased ammonia concentration, whereas the inclusion of DF reduced ammonia concentration in the cecal and colonic digesta. This observation agrees with Bikker et al. [28], who reported that ileal and colonic ammonia concentration was higher in the digesta of pigs fed a high protein diet, but when DF (i.e., sugar beet pulp and wheat middlings) was added, ammonia concentration in the digesta was reduced. Other studies have reported similar effects of high protein diets on digesta ammonia and other biogenic amine (putrescine, spermidine, spermine etc.) concentrations in the gut and the counteracting effect of the addition of a high fibre source [1,25]. Digesta SCFA and BCFA are considered key indicators of DF and protein fermentation in the gut and are generally considered to have positive and negative impacts on gut health, respectively. In the present study, we observed that high DF did not exert any significant independent effects on the concentration of fibre fermentation metabolites (e.g., acetic acid, butyric acid, and propionic acid) in the gut; however, the effects of fibre were evident in reducing the concentrations of BCFA (e.g., isobutyrate and isovalerate). The HfCP diet increased the concentration of BCFA in the cecal digesta while the inclusion of high DF generally reduced digesta BCFA concentration. The observations in the current study largely confirm a previous study indicating the role of DF as an alternate substrate for fermentation in high protein diets [26].

Under normal health conditions, organisms are protected against oxidation of tissues through antioxidant systems [28]. In situations of antioxidant deficiency, concentrations of oxidants are high, and tissues undergo oxidative stress [28]. Fermentation metabolites from DF and protein substrates (e.g., ammonia and SCFA) can increase oxidative stress in pigs and can have negative consequences on intestinal health and function [29]. The increase in serum antioxidant capacity with increasing dietary Thr observed in the current study is consistent with Azzam et al. [30], who demonstrated that increasing dietary Thr increased serum antioxidant capacity in laying hens. This indicates that, while antioxidant defense systems may not be impacted by DF and fCP, dietary Thr may be a limiting factor for optimal antioxidant capacity. Gene expression analysis showed significant treatment effects on target genes only in the colon. In the colonic tissue, feeding the HfCP diet reduced the expression of MUC2 gene, with no effect on MUC5ac, while feeding high DF increased the expression of both MUC2 and MUC5ac genes, which agrees with previous reports [14,31,32]. Expression of MUC2 was previously shown to be correlated with mucin secretion [31] which increases Thr requirements for protein deposition [2]. Therefore, this observation provides further support for the increase and decrease in Thr requirement for PD observed in the current study with high DF and high fCP diets, respectively. Reduced mucin secretion, and the associated barrier function, in high fCP diets may indicate a potential mechanism for greater incidence of pathogen-associated diarrhea in weanling pigs. High protein diets have been reported previously to negatively affect tight junction protein assembly and epithelial transport in pigs [33]. We observed in the present study that, feeding HF diet increased tight junction protein ZO-1expression in the LfCP but not the HfCP diet, which is indicative of the effects of high protein diets on tight junction assembly. Again, with a significant fibre × Thr interaction, we observed that ZO-1 expression increased as Thr increased in the LF, but not the HF fed pigs, indicating that the impact of Thr on ZO-1 expression is dependent on fibre level in the diet. On the other hand, the fCP × Thr interaction observed suggests that increasing Thr increased ZO-1 expression in the HfCP fed pigs but not in the LfCP fed pigs. These observations further prove that Thr utilization could be conserved and prioritized for maintaining intestinal integrity and function [22,34].

Previous studies have reported a reduced efficiency of utilizing dietary Thr for PD with increasing DF content, thereby increasing the dietary Thr requirement growth [2,8,9]. This effect is largely attributed to increased endogenous protein/AA losses [7,13,32]. In the present study, we observed that the HF diet reduced ATTD of N, which is consistent with reports from previous studies [2,8], but no significant effect of fCP was observed. Again, HF diet increased fecal N output and reduced urinary N output, which agrees with previous studies demonstrating a shift in N output from urine to feces which may be due to increased incorporation of N into microbial biomass [35]. The interactive association between DF and fCP on PD indicates that HF increased PD in the LfCP-fed but not the HfCP fed pigs. This observation is quite novel, as previous work has shown that feeding high DF will reduce growth performance when dietary Thr level is lower than requirement [8,9]. The negative effects of feeding high DF may be more evident when there is high fCP in the diet, as observed in the present study.

Within the LfCP fed pigs, HF increased PD compared to the LF fed pigs (165.4 vs. 154.7 g/d); however, in the HfCP group, HF did not increase PD compared to LF (173 vs. 176 g/d). This observation may suggest a possible negative physiological impact of combining HF and HfCP on the gut. Furthermore, when estimating the Thr requirement for maximum PD, we observed that, within the LfCP diet, feeding HF increased the Thr requirement for PD (0.73%, SID) compared to the LF diet (0.68%, SID). The same effect of DF was observed in HfCP-fed pigs, where inclusion of HF increased (0.64% SID) Thr requirement estimate for PD, compared to the estimate of 0.60% SID when fed an LF diet. However, while feeding high DF consistently increased the Thr requirement for PD, feeding a high fCP diet reduced the Thr requirement for PD. This observation agrees with previous studies which demonstrated an increased Thr requirement for PD [2], and growth performance [8,9] with the inclusion of high DF in the diet; however, the reduced Thr requirement in HfCP-fed pigs was unexpected. Previous studies have demonstrated the negative effects of high fCP on the intestinal epithelium and the subsequent effect on growth and nutrient metabolism [15,36]. It was our expectation that the negative effects of HfCP, as indicated by the parameters measured in the current study, would increase Thr requirements for PD; however, this was not the case. It is possible that the HfCP diet contained more available Thr than the LfCP diet; however, analysis of the diets showed no differences in total or SID dietary Thr across diets. Another possible reason for this observation could be the ages of pigs (>25 kg) used in the present study, which deviate from previous studies examining the impact of high protein diets. In the majority of these studies, these effects are studied in the immediate post-weaning period, during which pigs may be more susceptible to the negative effects of high protein diets on gut health (e.g., increased post-weaning diarrhea) [11,12]. Finally, it is also possible that the primary effect of fCP is not on dietary Thr and instead on another dietary factor, such as another AA, and, therefore, dietary Thr was not first limiting in the HfCP diets used in the current study. Overall, the HfCP diet showed negative effects on the gut; however, it did not result in increased dietary Thr requirement for PD, and the reason for this remains unclear.

## 5. Conclusions

In conclusion, the present study demonstrates that dietary fCP has a negative effect on intestinal health, as demonstrated by an increase in digesta ammonia and BCFA concentration and reduced expression of MUC2 gene, which is mitigated by the inclusion of DF. Further, the current study confirmed that high DF increases the Thr requirement for PD, while high fCP reduces Thr requirement for PD, regardless of the DF content. Overall, the inclusion of DF can be used to reduce the negative effects of high fermentable dietary protein content; however, dietary Thr content may need to be adjusted.

## Figures and Tables

**Table 1:** Composition of basal experimental diets (as-fed basis) ^1^.

Ingredient (%)	Low Fibre		High Fibre	
	LfCP	HfCP	LfCP	HfCP
Corn	43.96	34.31	26.63	20.52
Wheat	40.00	40.00	40.00	40.00
Canola oil	1.00	2.00	3.40	4.50
Soybean meal	10.00	-	10.00	-
Soybean meal (autoclaved) ^2^	-	20.00	-	16.00
Wheat bran	-	-	5.00	5.00
Sugar beet pulp	-	-	10.00	10.00
L-Lysine-HCl, 78.8%	0.87	0.59	0.85	0.69
DL-Methionine	0.13	0.09	0.14	0.12
L-Threonine, 98.5%	0.13	0.00	0.14	0.06
L-Tryptophan	0.06	0.01	0.05	0.02
L-Isoleucine	0.15	-	0.15	0.06
L-Leucine	0.16	-	0.25	0.10
L-Valine	0.22	0.05	0.23	0.13
L-Histidine	0.07	-	0.08	0.03
L-Phenylalanine	0.11	-	0.13	0.02
Limestone	1.20	1.20	1.00	1.00
Monocalcium phosphate	1.20	1.00	1.20	1.00
Salt	0.15	0.15	0.15	0.15
Vitamin-mineral premix ^3^	0.20	0.20	0.20	0.20
Celite	0.40	0.40	0.40	0.40
Calculated nutrient content ^4^				
DM (%)	88.3	87.4	86.0	85.0
CP (%)	15.8	18.8	16.1	17.8
Fermentable CP ^5^ (%)	2.04	2.79	2.38	3.33
ME (kcal/kg)	3286	3311	3303	3343
NE (kcal/kg)	2520	2500	2500	2513
Ca (%)	0.70	0.70	0.71	0.69
P (%)	0.60	0.60	0.61	0.60
SID Ile (%)	0.61	0.62	0.60	0.61
SID Leu (%)	1.17	1.25	1.17	1.17
SID Lys (%)	1.16	1.15	1.15	1.15
SID Met (%)	0.33	0.33	0.33	0.33
SID Cys (%)	0.24	0.27	0.25	0.26
SID Phe (%)	0.69	0.77	0.69	0.69
SID Thr (%)	0.52	0.52	0.52	0.52
SID Trp (%)	0.20	0.20	0.19	0.19
SID Val (%)	0.75	0.75	0.75	0.75

HfCP = high fermentable crude protein; LfCP = low fermentable crude protein; ME = metabolizable energy; NE = net energy; SID = standardized ileal digestible. ^1^ The basal Thr diet within each treatment (LF-HfCP, HF-HfCP, HF-LfCP and HF-HfCP) category is presented. The highest Thr content (not shown) were prepared by including L-Thr at the expense of corn in each basal diet, and the other graded Thr levels were prepared by blending the lowest and highest Thr diets within each treatment category in appropriate proportions. ^2^ Soybean was autoclaved at 124 °C for 20 min. ^3^ Supplied per kg of complete diet; vitamin A, 8 000 IU; vitamin D, 1 500 IU; vitamin E, 30 IU; menadione, 2.5 mg; vitamin B_12_, 0.025 mg; thiamine, 1.00 mg; biotin, 0.10 mg; niacin, 20 mg; riboflavin, 4 mg; pantothenate; 12 mg; folic acid, 0.50 mg; pyridoxine, 2.0 mg; Fe, 100 mg; Zn, 100 mg; Mg, 40 mg; Cu, 15 mg; Se, 0.30 mg; I, 1 mg. ^4^ Nutrient content of diets based on nutrient content of feed ingredients according to NRC [16]. ^5^ Calculated as CP—(total CP × SID CP). The SID of autoclaved soybean meal was estimated according to Gonzalez-Vega et al. [17].

**Table 2:** Dietary treatment effects on protein deposition ^1,2^.

SID Thr:	LfCP					HfCP					SEM	*p-*value				
	0.52	0.60	0.68	0.76	0.82	0.52	0.60	0.68	0.76	0.82		Fibre	Thr	fCP	Fib × fCP	Thr × fCP
Nitrogen intake (g/d)																
LF	35.4	34.1	34.5	35.8	36.1	41.1	45.0	41.2	41.5	41.5	1.09	0.031	0.025	<0.001	<0.001	0.043
HF	37.7	38.2	37.8	37.3	39.1	39.6	41.2	41.4	38.7	43.4						
Urinary nitrogen output (g/d)																
LF	3.94	2.77	3.86	4.08	3.13	6.61	5.50	4.99	5.97	5.27	0.65	<0.001	0.082	<0.001	0.011	0.699
HF	3.21	2.24	2.65	2.42	3.12	3.61	3.01	3.26	4.29	3.92						
Fecal nitrogen output (g/d)																
LF	6.93	6.47	5.75	7.21	7.86	8.53	8.57	7.78	8.11	8.41	0.54	<0.001	0.001	<0.001	0.027	0.334
HF	8.28	9.72	8.18	8.62	9.24	9.92	9.20	8.91	9.02	10.01						
ATTD of nitrogen (%)																
LF	80.1	81.1	83.5	79.9	78.1	79.1	80.9	81.1	80.5	79.7	1.23	<0.001	<0.001	0.235	0.175	0.081
HF	78.0	74.6	78.3	76.9	76.3	75.0	77.6	78.5	76.7	76.9						
Nitrogen retained (g/d)																
LF	24.5	24.9	24.9	24.5	24.9	25.9	30.9	28.4	27.4	24.8	1.16	0.083	0.004	<0.001	0.019	0.028
HF	26.2	26.2	26.9	26.3	26.7	26.1	28.9	29.3	25.4	29.5						
Protein deposition (g/d) ^3^																
LF	153.3	155.4	155.6	153.2	156.1	161.9	193.1	177.4	171.4	173.7	7.28	0.083	0.004	<0.001	0.019	0.028
HF	164.1	163.7	168.4	164.3	166.7	162.8	181.1	182.8	158.6	184.2						

ATTD = apparent total tract digestibility; Fib = fibre; HF = high fibre; HfCP = high fermentable crude protein; LF = low fibre; LfCP = low fermentable crude protein; NS = not significant; fCP = fermentable crude protein; SID = standardized ileal digestible; Thr = threonine. ^1^ Values are least square means (*n* = 8/treatment). ^2^ There were no significant three-way interactions (*p* > 0.05). ^3^ Protein deposition calculated as N retained × 6.25.

**Table 3:** Short-chain (SCFA), branched-chain (BCFA) fatty acid and ammonia concentration in the cecal digesta ^1,2^.

Item	Standardized Ileal Digestible Threonine, %						SEM	*p-*Value			
	0.52	0.68	0.82	0.52	0.68	0.82					
	LfCP			HfCP				Fibre	Thr	fCP	Fibre × Thr
Acetic acid, μmol/g											
Low fibre	63.86	59.81	50.21	79.98	75.89	53.87	8.36	0.410	0.332	0.079	0.109
High fibre	62.33	49.81	59.79	58.44	63.78	65.53					
Propionic acid, μmol/g											
Low fibre	27.16	26.26	22.33	25.26	24.77	21.44	4.21	0.117	0.928	0.158	0.456
High fibre	29.48	30.28	33.73	24.17	26.75	25.99					
Isobutyrate, μmol/g											
Low fibre	0.23	0.13	0.10	0.55	0.29	0.12	0.12	0.044	0.562	0.245	0.071
High fibre	0.10	0.19	0.11	0.04	0.10	0.15					
Butyric acid, μmol/g											
Low fibre	11.91	9.22	7.32	10.29	13.12	8.35	1.74	0.515	0.909	0.576	0.028
High fibre	10.45	9.79	11.80	8.62	10.48	13.02					
Isovalerate, μmol/g											
Low fibre	0.33	0.21	0.18	0.69	0.25	0.16	0.12	0.069	0.493	0.407	0.006
High fibre	0.05	0.23	0.27	0.12	0.11	0.31					
Valeric acid, μmol/g											
Low fibre	1.86	1.53	1.44	2.55	1.98	1.14	0.86	0.135	0.978	0.624	0.360
High fibre	1.77	4.03	2.86	2.28	1.23	2.84					
Total SCFA, µmol/g											
Low fibre	104.92	97.08	81.35	118.16	116.18	85.05	11.57	0.087	0.481	0.425	0.525
High fibre	104.14	95.03	108.66	93.83	102.56	107.57					
Total BCFA, µmol/g											
Low fibre	43.67	42.25	41.60	52.55	49.96	44.83	3.56	0.049	0.654	0.031	0.229
High fibre	43.35	38.59	41.50	40.81	41.26	46.64					
Ammonia, mg/dL											
Low fibre	6.97	5.03	8.85	10.31	10.01	7.71	1.33	<0.001	0.283	0.005	0.855
High fibre	2.94	1.52	1.88	3.68	2.44	5.48					

BCFA = branched-chain fatty acids; HfCP = high fermentable crude protein; LfCP = low fermentable crude protein; NS = not significant; SCFA = short-chain fatty acids; fCP = fermentable crude protein. ^1^ Values are least square means (n = 8/treatment). ^2^ There was no significant main effect of threonine and no significant three-way interactions or other two-way interactions apart from what is reported.

**Table 4:** Short-chain (**SCFA**), branched-chain (**BCFA**) fatty acid and ammonia concentration in the colonic digesta ^1^.

Item	Standardized Ileal Digestible Threonine, %						SEM	*p-*Value		
	0.52	0.68	0.82	0.52	0.68	0.82				
	LfCP			HfCP				Fib	Thr	fCP
Acetic acid, μmol/g										
Low fibre	45.67	51.93	44.51	38.59	47.83	37.97	7.03	0.104	0.484	0.303
High fibre	50.63	50.47	55.76	55.21	53.06	41.24				
Propionic acid, μmol/g										
Low fibre	18.73	19.24	13.88	14.30	15.06	13.82	2.65	0.913	0.143	0.043
High fibre	16.84	19.13	16.19	13.34	17.00	11.51				
Isobutyrate, μmol/g										
Low fibre	0.62	0.83	0.99	0.79	0.88	0.58	0.13	0.015	0.338	0.926
High fibre	0.39	0.71	0.63	0.66	0.43	0.74				
Butyric acid, μmol/g										
Low fibre	9.07	9.12	10.68	7.84	8.81	7.5	1.59	0.846	0.996	0.216
High fibre	10.42	8.22	9.46	8.22	9.49	8.27				
Isovalerate, μmol/g										
Low fibre	0.81	1.06	1.31	1.02	1.13	0.72	0.18	0.011	0.554	0.974
High fibre	0.52	0.83	0.74	0.86	0.56	0.96				
Valeric acid, μmol/g										
Low fibre	2.65	2.34	2.21	1.9	1.75	1.41	0.43	0.063	0.777	0.062
High fibre	1.24	2.18	1.64	1.49	1.35	1.52				
Ammonia, mg/dL										
Low fibre	11.68	11.78	11.08	15.42	15.72	13.73	1.93	0.002	0.492	0.016
High fibre	10.02	7.50	5.66	10.46	8.06	10.77				

BCFA = branched-chain fatty acids; fCP = fermentable crude protein; Fib = fibre; HfCP = high fermentable crude protein; LfCP = low fermentable crude protein; NS = not significant; SCFA = short-chain fatty acids; Thr = threonine. ^1^ There were no significant two-or-three-way interactions were observed (*p* > 0.05).

**Table 5:** Serum antioxidant concentration (Mm) ^1,2^.

SID Thr:	LfCP					HfCP					SEM	*p-*Value		
	0.52	0.60	0.68	0.76	0.82	0.52	0.60	0.68	0.76	0.82		Fibre	Thr	fCP
Low fibre	0.37	0.37	0.34	0.37	0.39	0.28	0.45	0.33	0.37	0.38	0.03	0.366	0.025	0.960
High fibre	0.30	0.40	0.34	0.32	0.38	0.34	0.33	0.37	0.36	0.36				

HfCP = high fermentable crude protein; fCP= fermentable crude protein; LfCP = low fermentable crude protein; NS = not significant; SID = standardized ileal digestible. ^1^ Values are least square means (n = 8/treatment). ^2^ There were no significant two-or three-way interactions (*p* > 0.05).

**Table 6:** Colonic tissue mRNA abundance (arbitrary units) of markers of intestinal health in pigs ^1^.

SID Thr:	LfCP			HfCP			SEM	*p-*Value						
	0.52	0.68	0.82	0.52	0.68	0.82		Fibre	Thr	fCP	Fib × Thr	Fib × fCP	Thr × fCP	Fib × fCP × Thr
MUC2														
Low fibre	0.33 ^b^	0.51 ^ab^	0.49 ^ab^	0.54 ^ab^	0.39 ^b^	0.29 ^b^	0.08	0.196	0.349	0.001	0.260	0.015	0.662	0.001
High fibre	0.86 ^a^	0.44 ^b^	0.52 ^ab^	0.30 ^b^	0.37 ^b^	0.34 ^b^								
MUC5ac														
Low fibre	0.24	0.07	0.03	0.05	0.11	0.05	0.10	0.008	0.705	0.556	0.225	0.214	0.317	0.537
High fibre	0.12	0.19	0.29	0.19	0.47	0.28								
IL-10														
Low fibre	0.26 ^b^	0.19 ^b^	0.21 ^b^	0.23 ^b^	0.17 ^b^	0.16 ^b^	0.07	0.001	0.002	0.013	0.098	0.086	<0.001	<0.001
High fibre	0.73 ^a^	0.21 ^b^	0.31 ^b^	0.23 ^b^	0.23 ^b^	0.32 ^b^								
IL-1β														
Low fibre	0.02	0.02	0.12	0.84	0.78	0.33	0.11	0.003	0.020	<0.001	0.037	0.001	0.037	0.164
High fibre	0.02	0.29	0.03	0.12	0.62	0.12								
ZO-1														
Low fibre	0.18	0.18	0.23	0.19	0.33	0.32	0.06	0.022	0.199	0.915	0.002	0.013	0.006	0.264
High fibre	0.55	0.20	0.29	0.27	0.23	0.32								

fCP = fermentable crude protein; Fib = fibre; HfCP = high fermentable crude protein; LfCP = low fermentable crude protein; NS = not significant; SID = standardized ileal digestible; Thr = threonine. ^1^ Values are least square means (n = 8/treatment). ^a,b^ Superscripts are presented for the significant 3-way interactions. Within each column and row for each parameter, means with different superscripts differ at *p* < 0.05

## Supplementary Materials

The following are available online at https://www.mdpi.com/2076-2615/10/11/2055/s1, Table S1: Analyzed nutrient content (as-fed basis) of the experimental diets, Table S2: Primers used for quantitative PCR analysis.
