# Supplementary material for: Intestinal Health and Threonine Requirement of Growing Pigs Fed Diets Containing High Dietary Fibre and Fermentable Protein
Source: Animals (Basel). 2020 Nov 6;10(11):2055. doi: 10.3390/ani10112055 (PMC7694666; doi:10.3390/ani10112055)
Supplement: Supplementary file 1 [file animals-10-02055-s001.pdf]

Supplementary Material

Title: Intestinal health and threonine requirement of growing pigs fed diets containing fermentable fibre and fermentable protein

Author list: Michael O. Wellington, Rochelle B. Thiessen, Andrew G. Van Kessel, Daniel A Columbus

Table 1. Analyzed nutrient content (as-fed basis) of the experimental diets.

	Dietary Threonine (SID %)[1]				
	0.52	0.60	0.68	0.76	0.82
Dry matter (%)					
LfCP[1]-LF[2]	91.74	91.82	91.63	91.36	91.68
LfCP-HF[3]	91.73	91.73	91.81	91.75	91.72
HfCP[4]-LF	91.76	91.76	91.53	91.90	91.54
HfCP-HF	92.07	91.83	91.93	91.84	91.44
Crude protein (%)					
LfCP-LF	15.14	15.43	15.36	15.15	15.30
LfCP-HF	15.99	15.73	15.95	16.16	16.14
HfCP-LF	17.56	18.28	18.03	18.18	17.43
HfCP-HF	16.86	17.38	17.73	16.75	18.13
Total threonine (%)					
LfCP-LF	0.59	0.66	0.73	0.84	0.90
LfCP-HF	0.65	0.65	0.75	0.85	1.00
HfCP-LF	0.62	0.70	0.76	0.82	0.97
HfCP-HF	0.64	0.72	0.76	0.85	0.97
Total lysine (%)					
LfCP-LF	1.31	1.26	1.28	1.26	1.28
LfCP-HF	1.28	1.31	1.28	1.31	1.28
HfCP-LF	1.24	1.23	1.23	1.23	1.23
HfCP-HF	1.26	1.34	1.25	1.26	1.34
Total methionine (%)					
LfCP-LF	0.26	0.27	0.30	0.28	0.27
LfCP-HF	0.25	0.22	0.36	0.36	0.28
HfCP-LF	0.27	0.28	0.27	0.30	0.27
HfCP-HF	0.22	0.26	0.30	0.33	0.34
Total leucine (%)					
LfCP-LF	1.18	1.24	1.17	1.22	1.32
LfCP-HF	1.27	1.21	1.22	1.26	1.27
HfCP-LF	1.27	1.24	1.21	1.25	1.31
HfCP-HF	1.22	1.22	1.27	1.21	1.26
Total isoleucine (%)					
LfCP-LF	0.61	0.63	0.60	0.64	0.65

LfCP-HF	0.66	0.61	0.61	0.65	0.66
HfCP-LF	0.65	0.60	0.60	0.63	0.64
HfCP-HF	0.60	0.60	0.67	0.60	0.68
Total valine (%)					
LfCP-LF	0.78	0.77	0.79	0.81	0.79
LfCP-HF	0.76	0.79	0.81	0.84	0.80
HfCP-LF	0.80	0.75	0.76	0.77	0.78
HfCP-HF	0.78	0.79	0.78	0.78	0.81
Insoluble dietary fibre (%)					
LfCP-LF	11.8	10.9	11.0	11.2	11.5
LfCP-HF	15.7	16.4	16.0	16.3	15.0
HfCP-LF	11.1	11.4	11.4	11.6	11.6
HfCP-HF	16.7	16.9	16.2	16.4	16.4
Soluble dietary fibre (%)					
LfCP-LF	1.4	1.2	1.7	1.7	1.5
LfCP-HF	3.4	3.2	2.6	4.0	3.9
HfCP-LF	2.1	1.8	1.9	1.6	2.2
HfCP-HF	3.4	3.1	3.1	3.3	3.3
Total dietary fibre (%)					
LfCP-LF	13.2	12.1	12.8	12.9	12.9
LfCP-HF	19.1	19.6	18.6	20.4	18.9
HfCP-LF	13.2	13.2	13.3	13.2	13.8
HfCP-HF	20.1	20.0	19.3	19.7	19.8

HF = high-fibre; HfCP = high fermentable crude protein; LF = low fibre; LfCP = low fermentable crude protein; SID = standardized ileal digestible

[1]Calculated total threonine content was 0.63, 0.71, 0.79, 0.87, and 0.93% for the 0.52, 0.60, 0.68, 0.76, and 0.82% SID Thr diets, respectively

Table S2: Primers used for the quantitative PCR analysis

Gene	Forward [5′ to 3′]	Reverse [5′ to 3′]	Annealing Temperature, °C	NCBI accession number
RPL19	AACTCCCGTCAGCAGATCC	AGTACCCTTCCGCTTACCG	60	AF_435591
MUC2	ACCCGCACTACGTCACCTTC	GGCAGGACACCTGGTCATTG	62	BX671371
MUC5AC	CCCCGTCGAACCAAAGCA	GGGAGTACATGGAGGCGGT	60	XM_021082583
GAPDH	CTTCACGACCATGGAGAAGG	CCAAGCAGTTGGTGGTACAG	63	AF017079
ZO-1	ACGGCGAAGGTAATTCAGTG	CTTCTCGGTTTGGTGGTCTG	60	XM_003353439.2
IL-10	CCATGGAAGTGGTCCGCCAA	GCCCAGGTAGCCATGGATC	55	NW_003535218.1
IL-1β	AGAAGAGCCCATCGTCCTTG	GAGAGCCTTCAGCTCATGTG	62	NM_001005149

GAPDH = glyceraldehyde 3-phosphate dehydrogenase; IL-10 = Interleukin-10; IL-1β = Interleukin-1β; MUC2 = Mucin-2; MUC5AC = Mucin 5 (pair 275); RPL19 = ribosomal protein-L19; ZO1; Zonula Occludin -1
